# Serological Biomarkers and Diversion Colitis: Changes after Stimulation with Probiotics

**DOI:** 10.3390/biom11050684

**Published:** 2021-05-02

**Authors:** Ángela Rodríguez-Padilla, Germán Morales-Martín, Rocío Pérez-Quintero, Juan Gómez-Salgado, Carlos Ruiz-Frutos

**Affiliations:** 1 Department of General Surgery, Infanta Elena University Clinical Hospital, 21080 Huelva, Spain; angela.rodriguez.padilla@gmail.com (Á.R.-P.); german.dr@hotmail.com (G.M.-M.); 2 Department of General Surgery, Juan Ramón Jiménez University Clinical Hospital, 21005 Huelva, Spain; roc14589@hotmail.com; 3 Department of Sociology, Social Work and Public Health, Faculty of Labour Sciences, University of Huelva, 21007 Huelva, Spain; frutos@uhu.es; 4 Safety and Health Postgraduate Programme, Universidad Espíritu Santo, 092301 Guayaquil, Ecuador

**Keywords:** diversion colitis, probiotics, efferent loop stimulation, inflammatory bowel disease, C-reactive protein, Glasgow Pronostic Score, serological biomarkers

## Abstract

Diversion colitis is a non-specific inflammation of a defunctionalised segment of the colon after a temporary stoma has been performed. This inflammation is associated with an alteration of certain inflammatory serum markers. The aims of this study were, firstly, to evaluate the modification of inflammatory biomarkers after stimulation with probiotics prior to closure of the protective ileostomy. Secondly, to identify if a relationship could be established between the severity of diversion colitis and the alteration of inflammatory biomarkers in the blood. A prospective, randomized, double-blind, controlled study was conducted. Patients who underwent surgery for colorectal carcinoma with protective ileostomy between January 2017 and December 2018 were included, pending reconstructive surgery and with diversion colitis as diagnosis. The sample was randomly divided into a group stimulated with probiotics (SG) (*n* = 34) and a control group (CG) (*n* = 35). Histological and endoscopic changes were evaluated after stimulation, after restorative surgery and during the short-term follow-up after surgery, including the correlation with pro-inflammatory biomarkers in blood. As main findings, a significant decrease in C-reactive protein (CRP), Neutrophil/lymphocyte ratio (NLR ratio), and monocyte/lymphocyte ratio (LMR ratio) was observed in the SG versus the CG with a *p* < 0.001. A significant increase in transferrin values and in the platelet/lymphocyte ratio (PLR) was observed in the SG versus CG after stimulation with probiotics with a *p* < 0.001. A normalisation of CRP and transferrin levels was observed in the third month of follow-up after closure ileostomy, and NLR, LMR and PLR ratios were equal in both groups. Decreased modified Glasgow prognostic score was found in SG compared to CG after probiotic stimulation (*p* < 0.001). The endoscopic and histological severity of diversion colitis is associated with a greater alteration of blood inflammatory biomarkers. The stimulation with probiotics prior to reconstructive surgery promotes an early normalization of these parameters.

## 1. Introduction

Diversion colitis (DC) is an inflammation produced in a defunctionalised segment of the colon after a temporary stoma has been performed [1]. Described by Glotzer et al. in 1981 [2], it is characterized by endoscopic findings such as mucosal friability, oedema, erythema, appearance of polyps, ulcers, stenosis, and microscopic findings such as lymphoid follicular hyperplasia, infiltration of the lamina propria by lymphocytes, eosinophils, the appearance of plasma cells, architectural disruption, and the appearance of crypt abscesses [1]. Chronic inflammation produces an increase of serum biomarkers, like other systemic inflammatory diseases such as gastrointestinal tumour, systemic lupus erythematosus, inflammatory bowel disease and cardiovascular disease [3,4,5,6]. The Glasgow Pronostic Score (GPS) and its modified scale (mGPS) were used to quantify the inflammatory state, based on serum levels of C-reactive protein (CRP) and albumin [7]. High levels of CRP, acute phase reactant, and low levels of albumin, related to malnutrition and intestinal disorders, reflect a systemic inflammatory response. These can be used as prognostic predictors, already available in daily practice [8], together with other inflammatory response biomarkers (IRB) such as transferrin, the neutrophil/lymphocyte ratio (NLR), the platelet/lymphocyte ratio (PLR) and the lymphocyte/monocyte ratio (LMR).

The definitive treatment for diversion colitis is the restoration of bowel continuity [1,9]. Pharmacological treatments using instillations with short-chain fatty acids, mesalazine fiber or corticosteroids are reserved for patients who are not candidates for surgical treatment or for stimulation of the efferent loop prior to surgery [10,11,12]. Continuing on this line of research, the efferent loop has been stimulated through probiotics, which interact with the intestinal mucosa, decreasing the production of pro-inflammatory substances [13].

Probiotics are defined as live microorganisms that, when administered in adequate amounts, confer a health benefit on the host [13,14]. Probiotics and their metabolic products have been proposed as food supplements to achieve a healthier intestinal homeostasis and also as a treatment for pathologies with an important inflammatory component. Currently available probiotics, aimed at other pathologies with inflammatory conditions, produce a modulating effect in a transitory and limited way [15,16,17,18].

This study has two objectives. Firstly, to evaluate the modification of inflammatory biomarkers after stimulation with probiotics prior to closure of the protective ileostomy. Secondly, to identify if a relationship could be established between the severity of diversion colitis and the alteration of inflammatory biomarkers in the blood.

## 2. Materials and Methods

### 2.1. Study Design

This was a prospective, randomised, multicentre, double-blind experimental study comparing two groups of patients who underwent a surgery for colorectal carcinoma with protective ileostomy. The intervention group included patients treated with stimulation of the efferent loop with probiotics prior to transit reconstruction surgery; the control group was not treated with any substance.

### 2.2. Participants

#### 2.2.1. Sample Size

The sample size was calculated according to the cut-off values of inflammatory biomarkers ROC curves published in previous studies [3,4,5,6,7,8]. 50% of reduction/normalisation of pathological biomarkers (50 to 100%) was assumed. With an adjustment loss of 15%, 30 patients per group were required. 34 patients were recruited for the stimulated group (SG) and 35 patients for the control group (CG) for a confidence level of 95% and a power of 0.8.

#### 2.2.2. Selection of Patients

Between January 2017 and December 2018, all the patients from the three participating centres included in the surgical waiting list for temporary stoma closure after colorectal carcinoma were consecutively assessed to determine their inclusion in the study. The inclusion criteria were being over 18 years of age, disease-free having protective ileostomy after colorectal carcinoma surgery, with endoscopic and histological confirmation of diversion colitis and having signed the informed consent. The exclusion criteria being clinical history and histological confirmation of inflammatory bowel disease with colorectal involvement and refusal to participate in the study. Abandonment criteria were loss during follow-up, exitus, and anastomotic leakage after stoma closure.

**Figure 1:**
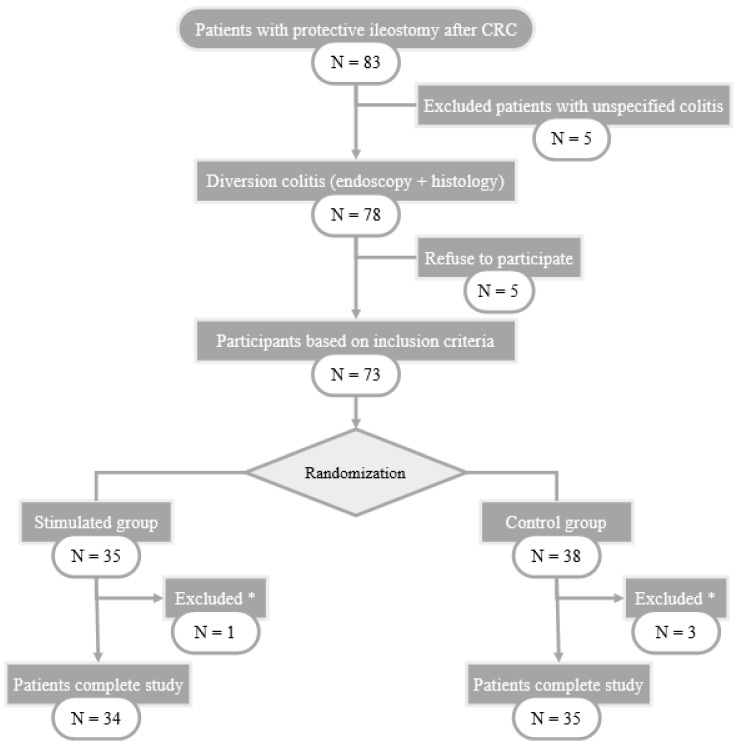
Flow-chart of patient selection. CRC: Colorectal Cancer; *: excluded patients with anastomotic leak.

During the study, 83 patients with protective ileostomy after colorectal carcinoma resection were assessed and included in the surgical waiting list for intestinal transit reconstruction. 78 of them met the endoscopic and histological criteria for diversion colitis diagnosis and 73 patients were finally randomized into two groups, intervention (*n* = 35) and control (*n* = 38). 69 patients completed the study, 1 of them from SG and 3 from CG abandoning the study because of anastomotic leakage. The selection flowchart of the study patients is shown in Figure 1.

#### 2.2.3. Randomization

A colonoscopy, including biopsies for histological study, was performed on all patients selected to participate in the study. After confirming the diagnosis, level of colitis (based on the Harig scoring system) [19], and excluding patients who did not meet the selection criteria, randomization was performed. Randomization was performed by using a computer-generated sequence (Statistical software EPIDAT version 4.2. Consellería de Sanidade, Xunta de Galicia, España; Organización Panamericana de la Salud (OPS-OMS); Universidad CES, Medellín, Colombia).

### 2.3. Data Collection

#### 2.3.1. Endoscopic and Histopathological Examination

Colonoscopies with random biopsies were performed on all patients to exclude those who did not have diversion colitis. Three endoscopists performed the endoscopies with a Silver Scope Storz^®^ colonoscope. The endoscopic findings were expressed according to the scoring system by Harig et al. [19], including erythema, oedema, friability, polyps, granularity, stenosis, and erosions. Every finding was scored in a scale from 0 to 1 or from 0 to 3, obtaining the colitis degree by adding them. The total score was categorised as absent, mild, moderate, or severe.

The histopathological analysis was assessed by three independent histopathologists. Samples of colon were fixed in buffered formalin and stained with Haematoxylin and eosin (H&E). In the same way as in the endoscopy, DC was diagnosed by evaluating the appearance of follicular lymphoid hyperplasia, eosinophilic, lymphocytes and plasma cells infiltrations and crypt architecture distortion. Every finding was scored in a scale from 0 to 1 or from 0 to 3, obtaining the grade of colitis by adding them. The total score was categorised as absent, mild, moderate or severe.

Both endoscopic and histopathological studies were performed after stimulation and 3 months after reconstruction of intestinal continuity.

#### 2.3.2. Determination of Serum Biomarkers

Blood extraction was performed. Basal levels of CRP and serum albumin were determined to calculate mGPS. A white blood cell count and transferrin determination were performed in a routine blood analysis in each hospital to establish the IRB, establishing cut-off values based on ROC curves.

These determinations were repeated after the completion of the stimulation phase, preoperatively, and in the third month of follow-up after surgery, correlating the pre and postoperative values with the endoscopic and histological alterations and persistence of diversion colitis.

#### 2.3.3. Intervention

Stimulation group (SG): preoperative stimulation of the distal limb of the ileostomy loop with probiotics was performed during the 20 days prior to surgery every second day. During and after the process, the patient himself registered the appearance of symptoms after each stimulation session: abdominal pain, emission of gas and stool. A sterile Foley catheter No.14 Ch connected to an infusion set was introduced through the defunctionalised bowel. This was done to allow slow infusion of a solution of 4.5 mg of probiotics diluted in 250 mL of 0.9% physiological saline for 20–30 min. Each preparation was made under sterile conditions and maintaining the cold chain. Vivomixx^®^ lyophilised live bacteria, marketed by MENDES, S.A, contained 4.5 × 10^11^ of live bacteria in each preparation:

∘ Four strains of *Lactobacillus*:
  ▪ *Lactobacillus acidophilus* DSM 24735^®^
  ▪ *Lactobacillus plantarum* DSM 24730^®^
  ▪ *Lactobacillus paracasei* DSM 24733^®^
  ▪ *Lactobacillus delbrueckii* subsp. bulgaricus DSM 24734^®^
∘ Three strains of *Bifidobacterium*:
  ▪ *Bifidobacterium breve* DSM 24732®
  ▪ *Bifidobacterium longum* DSM 24736®
  ▪ *Bifidobacterium infantis* DSM 24737®
∘ One strain of *Streptococcus:*
  ▪ *Streptococcus thermophilus* DSM 24731®

Control group (CG): exactly the same procedure was carried out, but the control group was stimulated without administering any substance, as the infusion set closed. During the process and after it, the patient himself registers the appearance of symptoms after each stimulation session: abdominal pain, emission of gas and stool.

After ten stimulation sessions, 24 h before surgery, a colonoscopy with biopsy was performed on all patients, re-quantifying the endoscopic and histological index of severity of diversion colitis. In the same way, blood was extracted to determine serum biomarkers.

### 2.4. Procedure

#### 2.4.1. Surgery and Follow-Up

All patients were admitted in the hospital the day before surgery, fasting, receiving antithrombotic prophylaxis (enoxaparin 40 mg subcutaneous) and premedication according to the pre-anaesthesia instructions sheet. The reconstruction surgery was carried out by three expert surgeons from the Colorectal Surgery department. A parastomal incision was made and carried out sharply into the peritoneal cavity. The anastomosis was lateral-lateral, either manual or mechanical, according to the decision of the surgeon. Every surgeon was allowed to decide whether to change to a median laparotomy procedure. Complications or events happened during surgery were recorded in the surgical procedure protocol. General anaesthesia was given to all patients and, after extubating and stabilization in the postoperative resuscitation room, they went directly to the hospitalization ward.

Follow-up during hospitalization was carried out by the staff of the Colorectal Surgery department of each centre, recording any postoperative complications, with special vigilance of abdominal pain, passage of flatus or stool with correct quantification and initiation of oral tolerance. Patients were discharged from the hospital after re-establishing intestinal transit, adequate oral tolerance and stool control, recording the length of stay in hospital.

Follow-up after hospitalization was carried out by the Colorectal Surgery team in the first and third postoperative months. These evaluations were performed by the colorectal surgeon who intervened in each patient. Any symptomatology related to the intervention was recorded, with special monitoring of abdominal pain and number and control of stools. After completing a 3-month follow-up, a colonoscopy with biopsies was performed to evaluate the presence and grade of DC. In the same way, blood was extracted to determine serum biomarkers.

#### 2.4.2. Blinding

To ensure blinding of the patients, all underwent the same diagnostic procedure. During the stimulation sessions, both the solution with probiotics and the infusion set were covered by an opaque protective envelope, which prevented observing the colour and transparency of the fluid, or whether the system was open or closed. The stimulation sessions were performed by a single surgeon, who was also in charge of preparing the dilution.

The endoscopist, the pathologist and the surgeon who performed the surgical intervention and the follow-up, as well as surgeons who participated, after the surgery, in the hospitalization process, did not know whether the patient had received probiotics or not.

#### 2.4.3. Assessment Criteria

The aim was to determine the relationship between diversion colitis and the increase of serum biomarkers and their modification after stimulation of the efferent loop with probiotics prior to closure of ileostomy in patients operated on colorectal carcinoma. The results were compared with the control group in the following situations: basal levels, after stimulation and at the third month of follow-up. The study also aimed to determine whether there is a relationship between the levels of severity of diversion colitis, both endoscopic and histological, and the alteration of pro-inflammatory biomarkers in the blood.

### 2.5. Statistical Analysis

A descriptive univariate analysis of sociodemographic and clinical variables was performed. The Kolmogorov-Smirnov test was used to verify the normality of the quantitative variables. To describe the quantitative variables, the mean and standard deviation were used, along with the median and interquartile range for those variables that did not follow a normal distribution. For qualitative variables, frequencies and percentages will be used.

The precision of each biomarker was evaluated by ROC (Receiver Operating Characteristic) curves to determine the optimal cut-off point for NLR, PLR, and LMR. Subsequently, a univariate test was performed to verify the main objectives. The box-plot graphics allowed to visualise the results in terms of response rates and CRP levels. Biochemical parameters were performed using the Fisher’s test or the chi-squared test for categorical variables, and the Mann–Whitney test for variables without normal distribution. Comparisons within each group to evaluate the modification of histological and endoscopic severity, CRP, and biochemical parameters were analysed using paired Wilcoxon and t-test. A *p* < 0.05 was considered to be significant. Statistical analyses were performed using the statistical programme SPSS^®^ version 24.0 (IBM, Armonk, NY, USA), with the support of calculation tools provided by the Microsoft Excel and R Commander software.

### 2.6. Ethical Aspects

The project was performed with the consent of the Ethics Coordinating Committee for Biomedical Research of Andalusia, Spain, and registered with the project number 2017/331191354. Written informed consent was requested to participate in the study, giving details of both the study objectives and the methodology to be followed. The data was kept anonymous, maintaining the confidentiality and anonymity of the participants.

## 3. Results

### 3.1. Sociodemographics

There were no significant differences between SG and CG in terms of sociodemographic, clinical or endoscopic and histological severity (Table 1).

### 3.2. Serological Biomarkers

**Figure 2:**
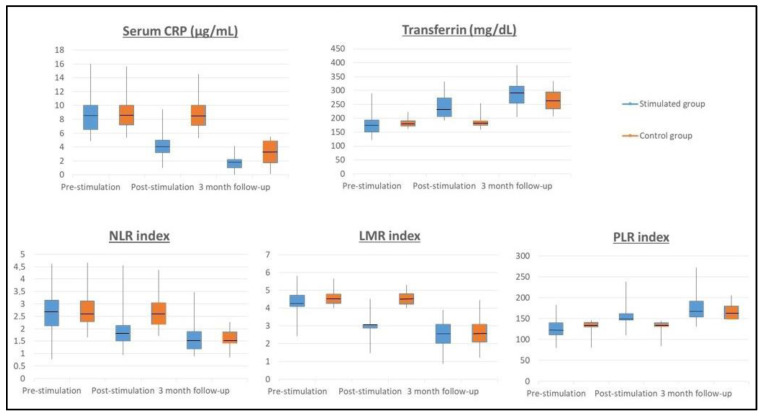
Inflammatory biomarkers throughout the study. Probiotics stimulation changes in serological biomarkers. The levels of CRP (C-reactive protein), NLR (neutrophil-to-lymphocyte ratio), LMR (lymphocyte-to-monocyte ratio), PLR (platelet-to-lymphocyte ratio), and transferrin were modified by probiotic consumption after the stimulation phase (*p* < 0.001). The levels of CRP, NLR, LMR, PLR, and transferrin were similar after 3-month follow-up.

The determination of the inflammatory markers was performed in different phases (Figure 2). No differences were found between SG and GC in the baseline determinations of CRP (*p* = 0.299), NLR (*p* = 0.457), LMR (*p* = 0.144), PLR (*p* = 0.150), or transferrin (*p* = 0.746).

A significant decrease in the CRP value was observed in the SG after completing the stimulation phase (pre-stimulation CRP = 7.82 µg/mL and post-stimulation CRP = 4.09 µg/mL) compared to the CG that maintains high CRP values (pre-stimulation CRP = 8.42 µg/mL and post-stimulation CRP = 8.28 µg/mL), *p* < 0.001. At the three-month follow-up, the CRP values normalized, with CRP levels of 2.9 µg/mL in the CG and 1.58 µg/mL in the SG. NLR count figures significantly decreased in the SG from pre-stimulation (NLR = 2.58) to post-stimulation (NLR = 1.72) *p* < 0.001, while the figures of NLR in CG remained high (pre-stimulation NLR = 2.74 and post-stimulation NLR = 2.75), *p* < 0.001. At the three-month follow-up, the NLR ratio normalised in both groups, with an NLR value of 1.46 in the CG and of 1.57 in the SG. Regarding the LMR count a significant reduction was found in the SG after completing the stimulation phase (pre-stimulation LMR = 4.22 and post-stimulation LMR = 3.24), *p* < 0.001. The CG remained high LMR ratio (pre-stimulation LMR = 4.62 and post-stimulation LMR = 4.42). At the three-month follow-up, the LMR ratio normalized in both groups, with LMR value of 2.40 in the CG and 2.66 in the SG. Result showed a significant increase in the PLR count in the SG after completing the stimulation phase from pre-stimulation PLR = 126.82 to post-stimulation PLR = 150.04, *p* < 0.001. Regarding the CG, the PLR score remained low (pre-stimulation PLR = 138.18 and post-stimulation PLR = 138.74). At the three-month follow-up, the PLR ratio normalized in both groups, with a PLR value of 161.54 in the CG and 167.67 in the SG.

Regarding transferrin values, they increased from 184 mg/dL to 238 mg/dL in the SG after completing the stimulation phase, but the CG maintained low transferrin values (pre-stimulation 181 mg/dL and post-stimulation 185 mg/dL), *p* < 0.001. At the three-month follow-up, transferrin values normalized, with 265 mg/dL in the CG and 289 mg/dL in the SG. The m-GPS was calculated in both groups without finding statistically significant differences in the baseline determination of both groups. The m-GPS in the CG was 1 in 65.7% of the patients (*n* = 23) and 2 in 34.3% (*n* = 12 patients), which figures that remained stable after stimulation. The m-GPS in the SG was 1 in 64.7% of the patients (*n* = 22) and 2 in 35.3% (*n* = 12 patients). After stimulation, the m-GPS in SG was 0 in 91.2% of the patients (*n* = 31) and 1 in 8.8% (*n* = 3 patients), finding a significant decrease compared to the CG, *p* < 0.001.

### 3.3. Endoscopic/Histological Severity and Serological Biomarkers

When comparing the mean values of the different serological determinations by histological and endoscopic severity in DC, we found that patients with high severity index had higher mean values in the determination of CRP, NLR, and LMR, and lower mean values in the determination of PLR and transferrin. Nonetheless, these differences were not statistically significant (Table 2). There were no differences regarding baseline determinations between SG and CG.

After the stimulation phase, the increase in CRP, NLR, and LMR values was maintained in CG, being higher in the most severe cases of DC, without finding statistically significant differences. Likewise, no significant differences were found when comparing PLR and transferrin associated with the most severe index of DC (Table 2). After the stimulation phase, a decrease in the mean values of CRP, NLR, and LMR was observed in the SG with moderate and mild severity index and absence of histological and endoscopic DC. Although these differences were not statistically significant when compared with each other, when they were compared with baseline levels, a *p* < 0.001 was obtained. Likewise, there was no statistically significant increase in the mean values of PLR and transferrin in the group of moderate and mild severity index and absence of histological and endoscopic DC, but statistically significant differences were found when compared with baseline levels, with a *p* < 0.001 (Table 2).

### 3.4. Safety and Adverse Effects

No serious complications were reported following the consumption of the probiotics in participants throughout the stimulation. Abdominal pain was the only symptom observed during stimulation, which was present in 20.5% of patients SG (*n* = 7) compared to 14.3% CG (*n* = 5) (no statistically significant differences). Abdominal pain was evaluated using the visual analog scale (VAS). Pain was moderated in SG, only present in the first stimulation sessions and disappearing afterwards. Pain was mild-moderate in CG in all stimulation sessions and disappeared after their completion.

## 4. Discussion

This is the first published randomized, double-blind study linking DC with alterations in inflammatory biomarkers. In this study, it was observed that serological pro-inflammatory biomarkers improved after stimulation with probiotics in SG compared to GC. The stimulation of the efferent loop with probiotics also improved the inflammation of the excluded colon mucosa, decreasing the inflammatory severity index and the inflammatory serum biomarkers. The differences for the studied biomarkers were statistically significant, *p* < 0.001. We observed a relationship between alterations in inflammatory biomarkers and endoscopic and histological severity index, although these findings were not statistically significant.

Several studies have been published about an alternative treatment to surgery, both in experimental models and phase III studies. The alternative to surgery consists in stimulation with different substances such as nutritional solutions, faecal transplantation, short-chain fatty acid (SCFA) enemas such as butyrate, acid 5 aminosalicylate (5-ASA), glucocorticoids, sucralfate and N-acetylcysteine among others, with different results [1,9,20,21,22,23,24,25,26,27,28,29,30,31,32]. Most of them showed inconsistent results, although there seems to be a decrease in intestinal mucosa inflammation, epithelial lesions and neutrophil infiltration. Nowadays the most successful treatment is the closure of the protective ileostomy. However, there are no studies that mention whether these patients or experimental animals concomitantly present increase of inflammatory biomarkers. These serological alterations have been described and studied in pathologies such as inflammatory bowel disease, seeking the correction of these inflammatory parameters associated with dysbiosis, also with variable results [15,16,17]. For this reason, our results are encouraging, since they demonstrate a reduction in inflammatory parameters prior to reconstruction of the transit, thus being a possible alternative for those patients who are not candidates for surgical treatment. The only side-effect was the appearance of colicky abdominal pain in 20.5% of the patients (*n* = 7), valued as moderate pain using the visual analog scale (VAS) and only associated with the first stimulation sessions, and disappearing afterwards. This effect has already been described in studies with stimulation of the efferent loop with short chain fatty acids [9,11,19].

The relationship between inflammatory biomarkers and probiotics has been studied in other pathologies such as acute diarrhoea in adults, inflammatory bowel disease, active pouchitis and irritable bowel syndrome, among others [13,15,32,33,34,35,36,37,38]. The most studied probiotic bacteria are Bifidobacterium and Lactobacillus spp. These two bacteria produce a decrease of pro-inflammatory molecules and an increase of molecules that inhibit inflammation, also protecting against oxidative stress in humans and demonstrating an important role in intestinal dysbiosis [23,39,40]. Currently, the evidence showed that probiotics have a positive effect on systemic and oxidative inflammation, especially at the gastrointestinal level. L. acidophilus, one of the probiotics administered during our stimulation, has demonstrated its role in inflammatory regulation in multiple experimental studies [27,28,37,38,39,40,41,42,43,44,45]. A randomized, controlled clinical trial conducted by Jafarnejad et al. [46] described the effect of supplementation with probiotics for 8 weeks, in this case, with VSL3 (a probiotic similar to the one administered in our study), on glycaemic status and inflammatory markers among pregnant women. The probiotic supplements contained eight strains of lactic acid bacteria (*S. thermophilus*, *Bifidobacterium breve*, *Bifidobacterium longum*, *Bifidobacterium infantis*, *L. acidophilus*, *L. plantarum*, *L. paracasei* and *L. delbrueckii subsp. Bulgaricus*). They found a statistically significant decrease in TNF alpha and CRP levels in the probiotic group compared to placebo [45]. Another clinical trial explained the efficacy of VSL3 probiotics administered in 24 patients with irritable Bowel Syndrome for 8 weeks, finding a significant improvement in symptoms, but without significant differences between the groups [47].

Finally, chronic inflammatory processes such as DC can raise CRP, NLR and LMR values (inflammatory markers), and decrease levels of albumin, transferrin and another inflammatory marker such as the PLR index [48,49]. In our study, the probiotics anti-inflammatory effect seemed to be demonstrated after the stimulation phase, where we observed a decrease of serological inflammatory parameters and normalization of CRP, PLR and transferrin values in the SG and a statistically significant decrease in NLR and LMR values, yet without normalization, with a *p* < 0.001. The normalization of the CRP, transferrin, NLR, LMR and PLR values was only achieved after reconstructive surgery and 3 months after surgery. These results could be explained by the ability of probiotics to interact with the intestinal mucosa, decreasing the molecular production of pro-inflammatory substances, and thereby decreasing the capacity for migration of inflammatory cells to the lamina propria, such as lymphocytes, eosinophils and plasma cells [31,32,33]. In addition, a correlation between high CRP values and an alteration in the percentage of lymphocytes in peripheral blood determinations has also been observed in previous studies [5]. Lymphocytes are the main components that infiltrate the mucosa and can range from mild to severe depending on the inflammation of the excluded colon segment in humans. However, in our study we did not find statistically significant differences between the increase of serum biomarkers and the severity index of DC.

Supporting our study results, it is worth noting that patients had a high adherence to the treatment in both groups, only excluding patients with postoperative complications, without registering other losses during follow-up. In addition, our study population only included patients who met endoscopic and histological diagnostic criteria for DC, excluding patients with a diagnosis of non-specific colitis despite presenting a protective ileostomy, since we considered that they could represent a misleading factor and lead to error.

## 5. Conclusions

It could be concluded that endoscopic and histological severity index of diversion colitis is related to a greater alteration of inflammatory biomarkers in the blood and that stimulation with probiotics prior to reconstructive surgery produces an earlier normalization of these parameters, making it an option for patients who are not eligible for surgical treatment.

## Figures and Tables

**Table 1:** Sociodemographic and clinical variables and endoscopic and histological severity index of diversion colitis between the stimulated group (SG) and the control group (CG).

	Stimulated Group (*n* = 34)	Control Group (*n* = 35)	*p*
Demographics			
Age (years)	65 (45–81)	68 (41–80)	0.421
Sex ratio (M:F)	23:11	25:10	0.170
BMI (kg/m^2^)	23.5 (21.6–32.6)	27.6 (18.8–40.2)	0.091
ASA			0.483
ASA I-II	31	30	
ASA III	3	5	
Smoker/non-smoker	20/14	23/12	0.826
Time between surgery (months) *	12 (8–37)	9 (6–32)	0.813
Clinic			
Asymptomatic	10 (29.4%)	14 (40%)	0.309
Abdominal pain	15 (44.1%)	18 (51.4%)	0.402
Tenesmus	5 (14.7%)	2 (5.7%)	0.702
Mucous Discharge	21 (61.7)	14 (40%)	0.117
Rectorrhagia	2 (5.9%)	4 (11.4%)	0.668
Endoscopic severity			
Mild	2 (5.9%)	3 (8.6%)	0.511
Moderate	23 (67.6%)	23 (65.7%)	0.648
Severe	9 (26.5%)	9 (25.7%)	0.498
Histological severity			
Mild	4 (11.8%)	3 (8.6%)	0.479
Moderate	21 (61.7)	23 (65.7%)	0.321
Severe	9 (26.5%)	9 (25.7%)	0.518

BMI: body mass index. ASA: American Society of Anesthesiologists Classification. * Time from creation of stoma to the closure of the protective ileostomy.

**Table 2:** Inflammatory markers according to endoscopic + histological severity index before and after stimulation.

Severity Index		Serum CRP					
		Stimulated Group (*n* = 34)			Control Group (*n* = 35)		
		*n* *	Me	RI	*n*	Me	RI
Pre-stimulation	Severe	9	11.03	±4.9	9	12.16	±3.14
	Moderate	21	10.51	±3.37	23	10.1	±1.96
	Mild	4	5.27	±0.37	3	7.83	±1.53
Post-stimulation	Severe	-	-		9	11.96	±2.91
	Moderate	3	7.35	±3.06	23	9.95	±2.09
	Mild	19	3.8	±1.72	3	7.67	±1.33
	Absent	12	4.2	±1.03	-	-	
		NLR ratio					
Pre-stimulation	Severe	9	3.17	±1.53	9	2.92	±0.65
	Moderate	21	3.01	±1.1	23	2.87	±0.32
	Mild	4	2.58	±0.94	3	2.62	±0.47
Post-stimulation	Severe	-	-		3	2.98	±0.73
	Moderate	3	1.97	±0.85	23	2.83	±0.30
	Mild	19	1.7	±0.46	3	2.54	±0.45
	Absent	12	2.39	±1.28	-	-	
		LMR ratio					
Pre-stimulation	Severe	9	4.82	±0.63	9	4.99	±0.31
	Moderate	21	4.24	±0.31	23	4.62	±1.47
	Mild	4	3.96	±1.17	3	4.44	±0.30
Post-stimulation	Severe	-	-		9	4.99	±0.57
	Moderate	3	3.75	±0.36	23	4.31	±0.27
	Mild	19	3.11	±0.71	3	4.36	±0.27
	Absent	12	3.72	±1.06	-	-	
		PLR ratio					
Pre-stimulation	Severe	9	114.74	±19.79	9	130.37	±19.27
	Moderate	21	121.87	±20.83	23	134.82	±9.31
	Mild	4	131.76	±3.56	3	142.08	±1.95
Post-stimulation	Severe	-	-		9	132.66	±18.08
	Moderate	3	154.50	±26.07	23	134.57	±8.16
	Mild	19	158.37	±3.56	3	140.99	±1.84
	Absent	12	153.61	±31.31	-	-	
		Serum Transferrin					
Pre-stimulation	Severe	9	174.66	±43.14	9	174.55	±10.51
	Moderate	21	184.96	±32.01	23	184.34	±10.09
	Mild	4	196.60	±17.53	3	211.66	±21.50
Post-stimulation	Severe	-	-		9	174.11	±9.08
	Moderate	3	248.66	±68.82	23	182.39	±11.05
	Mild	19	252.01	±42.32	3	217.33	±38.07
	Absent	12	232.25	±35.53	-	-	

To describe the quantitative variables, the median (Me) and interquartile range (RI) were used. CRP: C-reactive protein. NLR (neutrophil-to-lymphocyte ratio). LMR (lymphocyte-to-monocyte ratio). PLR (platelet-to-lymphocyte ratio). n * of the stimulated group changes because the stimulation with probiotics produces a decrease in the endoscopic and histological severity of diversion colitis.

## Data Availability

All data generated in this study is showed in this article, its tables, and figures.
